# Supplementary figures and images for: Characterization of a biofilm-forming Shigella flexneri phenotype due to deficiency in Hep biosynthesis
Source: PeerJ. 2016 Jul 14;4:e2178. doi: 10.7717/peerj.2178 (PMC4950558; doi:10.7717/peerj.2178)

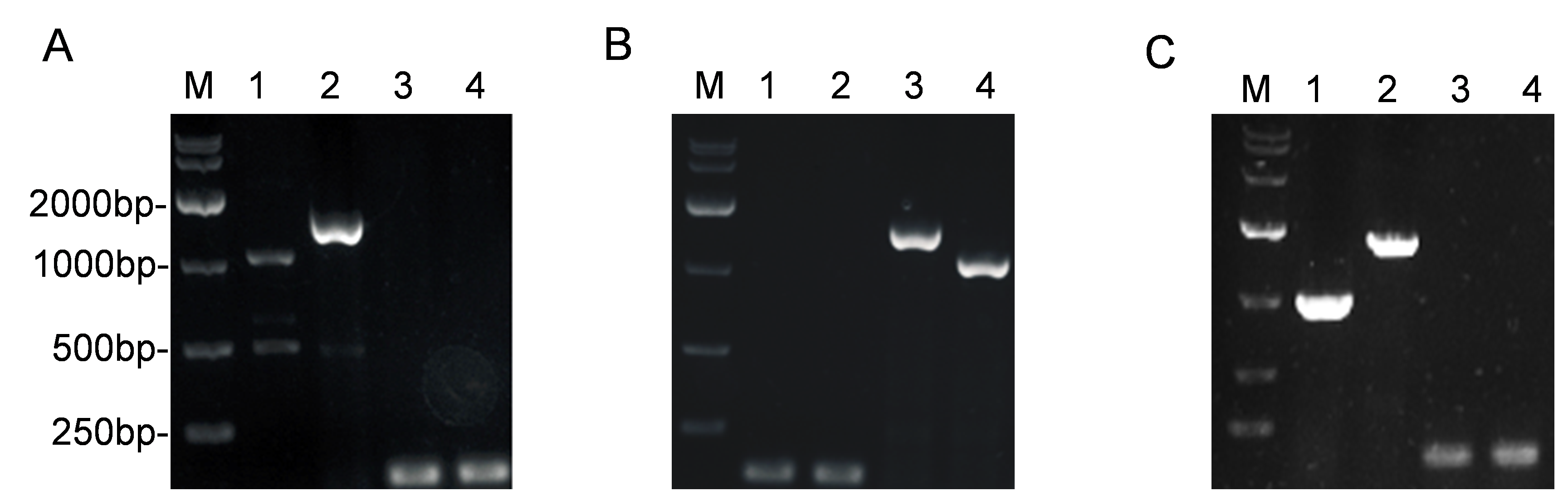

Supplement: Figure S1 — PCR was performed using the primers listed in Table 2. DNA ladders were shown in Lane M. (A) Δwzy mutant strain. Lane 1, wild type (1238 bp); Lane 2, Δwzy: kana (1600 bp); Lane 3, 4, Δwzy (100 bp); (A) ΔwaaL mutant strain. Lane 1, wild type (1238 bp); Lane 2, ΔwaaL: kana (1600 bp); Lane 3, 4, ΔwaaL (100 bp); (A) ΔrfaC mutant strain. Lane 1, wild type (1238 bp); Lane 2, ΔrfaC: kana (1600 bp); Lane 3, 4, ΔrfaC (100 bp). [file peerj-04-2178-s001.png]

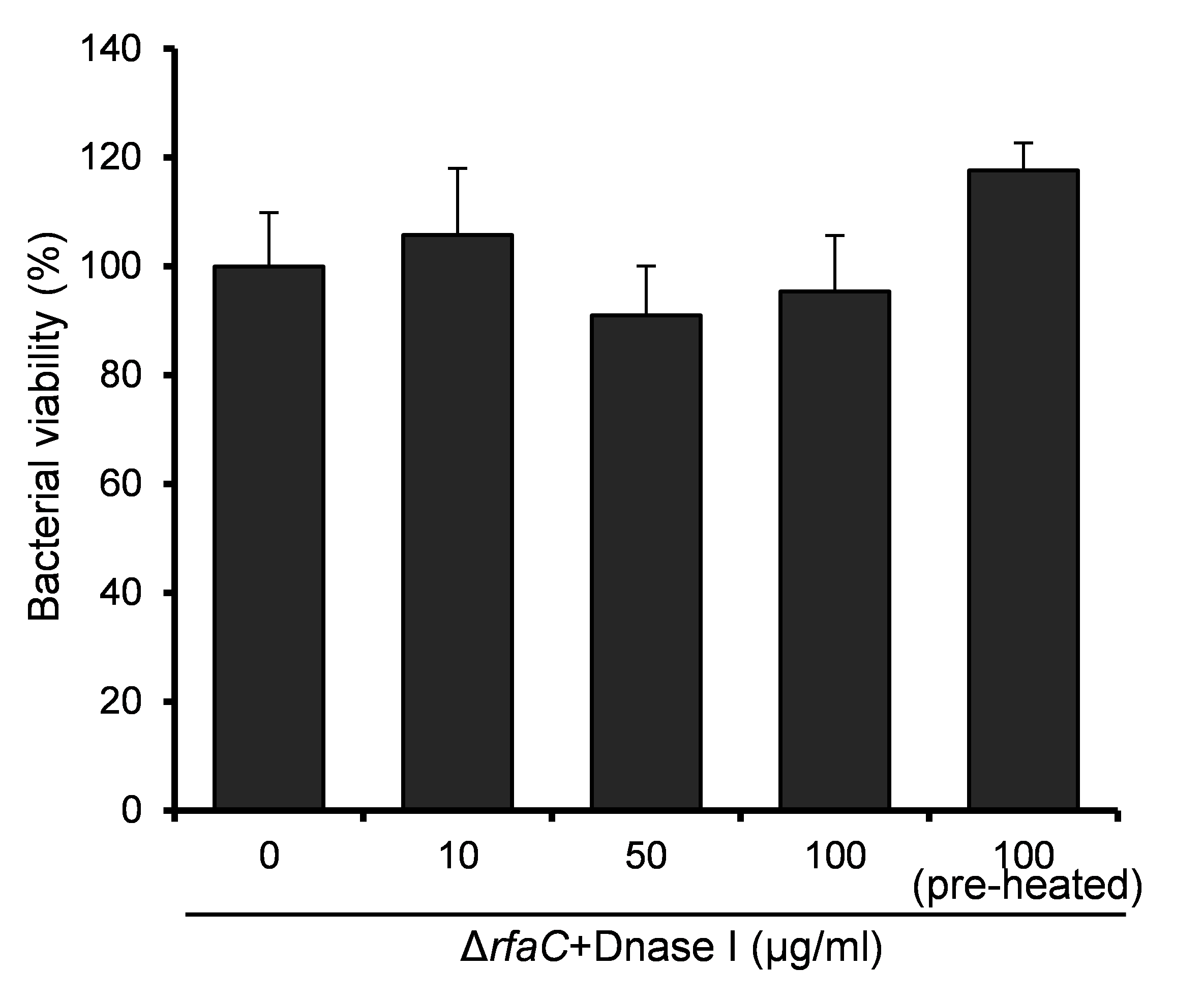

Supplement: Figure S2 [file peerj-04-2178-s002.png]

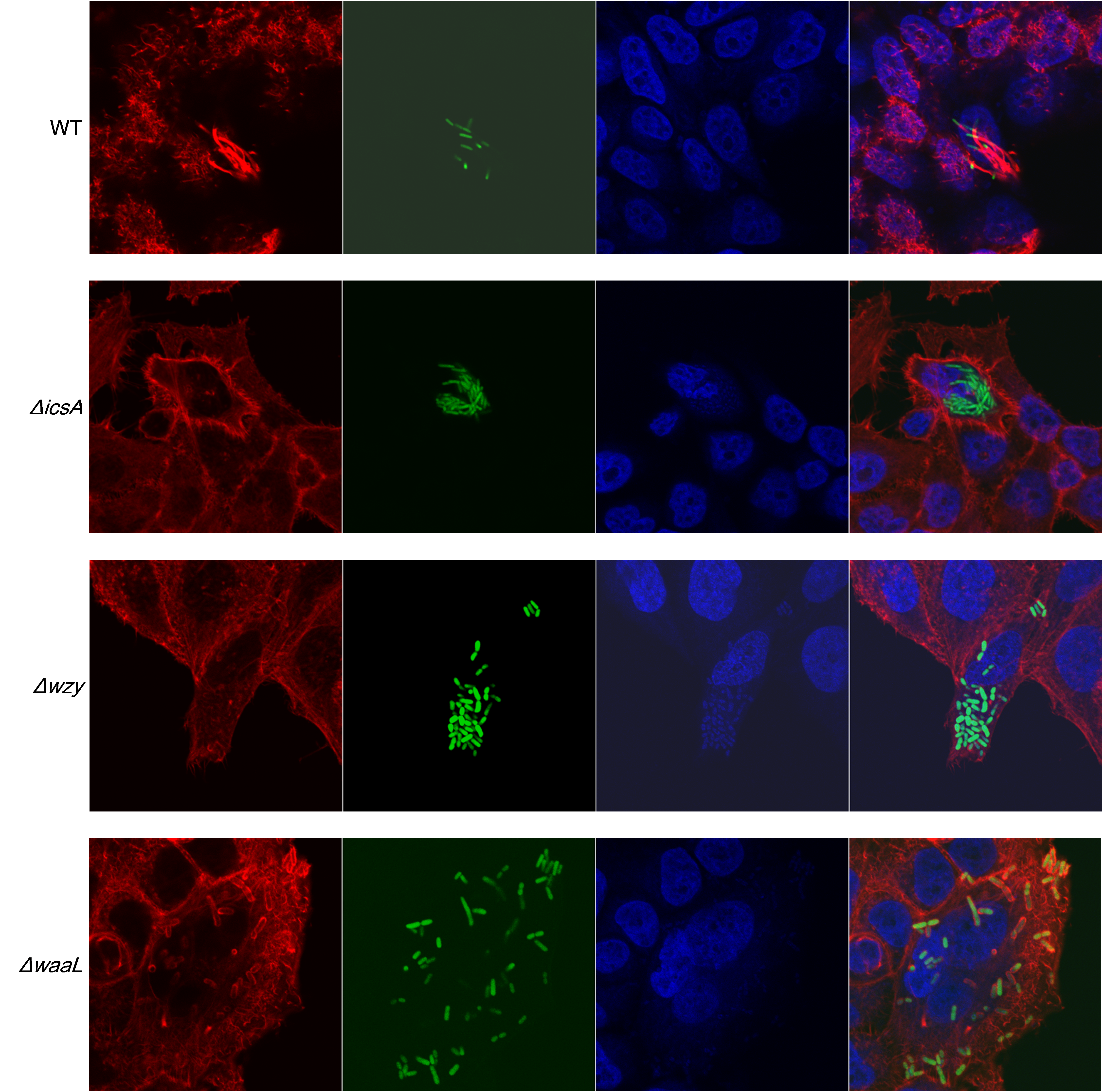

Supplement: Figure S3 — Invasion assay was performed as previously described. F-actin is stained by TRITC-phalloidin (red), Shigella bacteria are green and nuclei are blue (DAPI). [file peerj-04-2178-s003.png]
